# Supplementary figures and images for: Antagonism of BST2/Tetherin, a new restriction factor of respiratory syncytial virus, requires the viral NS1 protein
Source: PLoS Pathog. 2024 Nov 19;20(11):e1012687. doi: 10.1371/journal.ppat.1012687 (PMC11614281; doi:10.1371/journal.ppat.1012687)

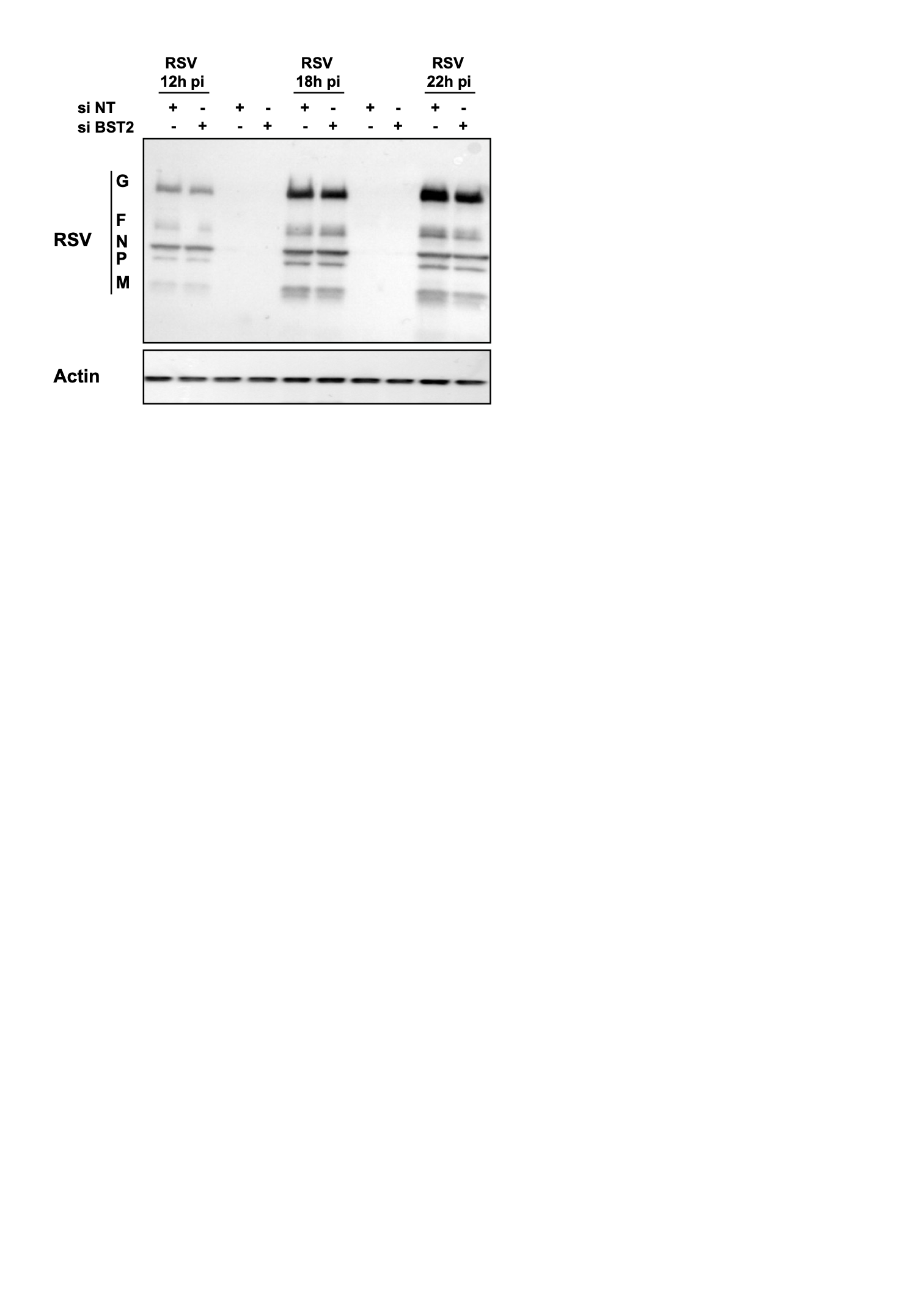

Supplement: S1 Fig — HEp-2 cells were infected with RSV virus at MOI of 1. Then, 12, 18 and 22h post infection, protein extracts were realized and processed for WB with RSV and actin antibodies (TIF) [file ppat.1012687.s001.tif]

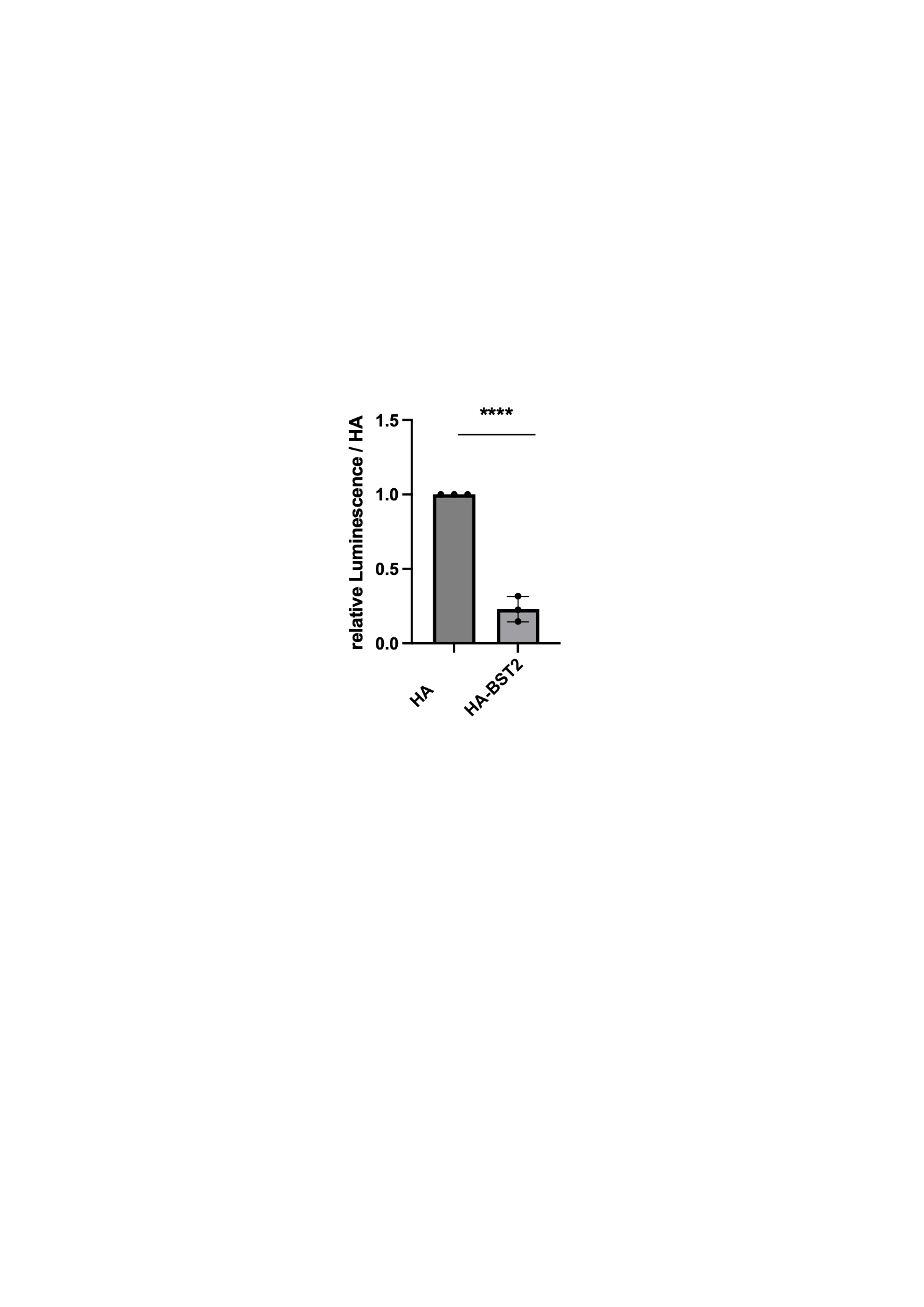

Supplement: S2 Fig — HEp–2 cells overexpressing HA-BST2 or HA were infected with RSV-Luc at MOI of 0,01. Then 48h post infection a luciferase assay was performed and data were expressed as a ratio of HA-BST2 cells on HA cells. 3 independent experiments with triplicates were performed and a nested t-test were performed **** p< 0.0001 (TIF) [file ppat.1012687.s002.tif]

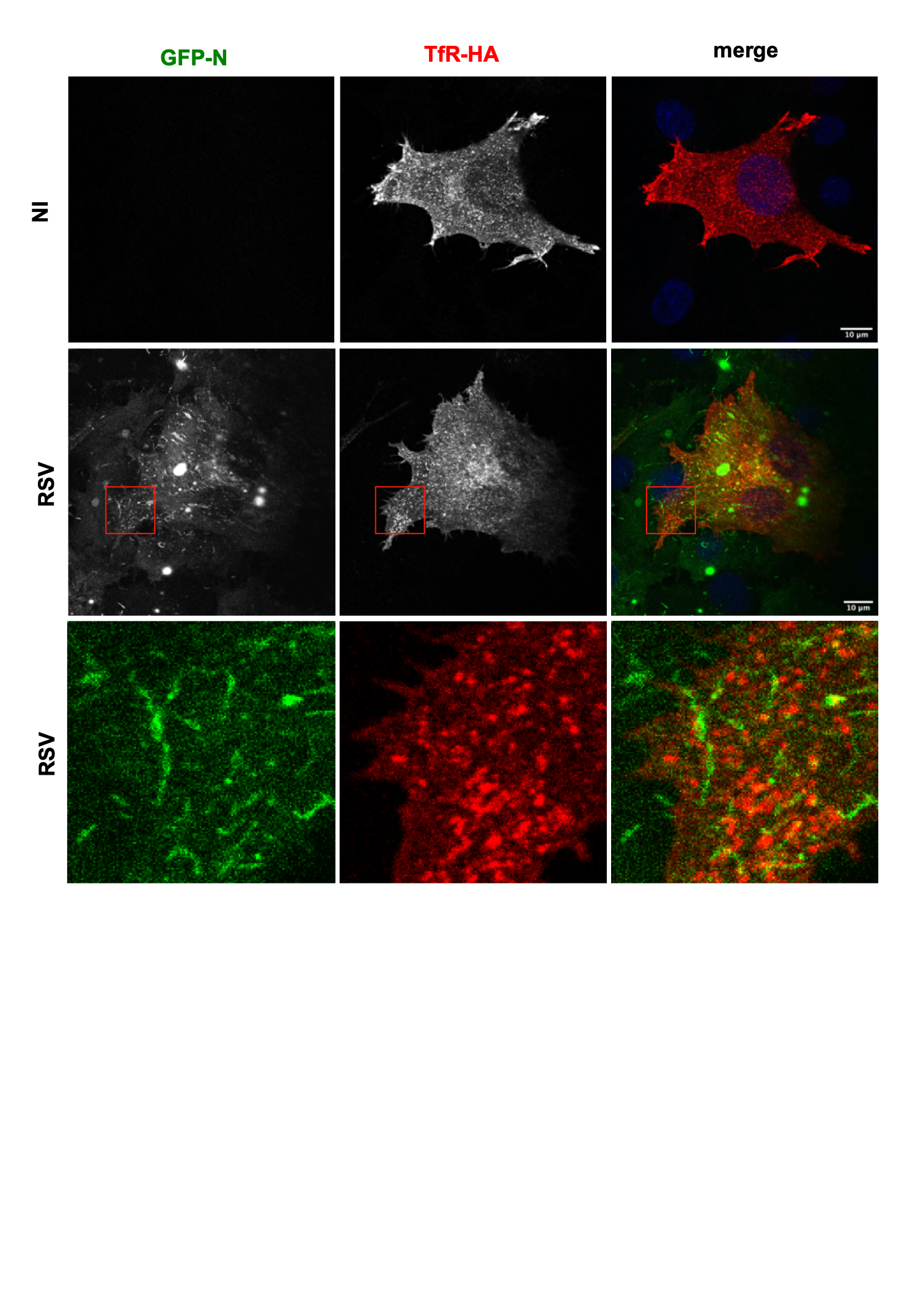

Supplement: S3 Fig — (A) HEp–2 cells overexpressing BST2 were infected or not with RSV-GFP-N. At 24h p.i. cells were fixed and stained with antibody against HA (red in merge). The GFP-N protein is visualized through its spontaneous green fluorescence (green in merge). Nucleus staining is shown in blue (merge). Representative images from 3 independent experiments are shown. NI (not infected). Zoom images of the red square are shown. Scale bar 10 μm. (TIF) [file ppat.1012687.s003.tif]

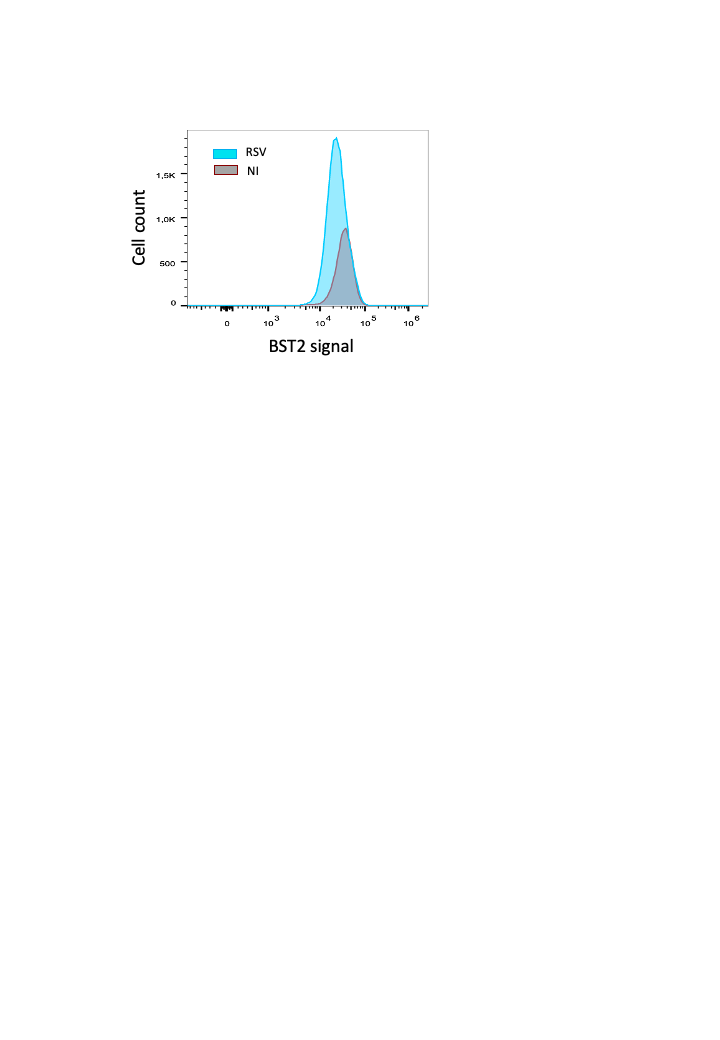

Supplement: S4 Fig — One representative FACS histogram of the bar graph from the Fig 3A. Non infected cells are depicted in grey and infected cells in blue. (TIF) [file ppat.1012687.s004.tif]

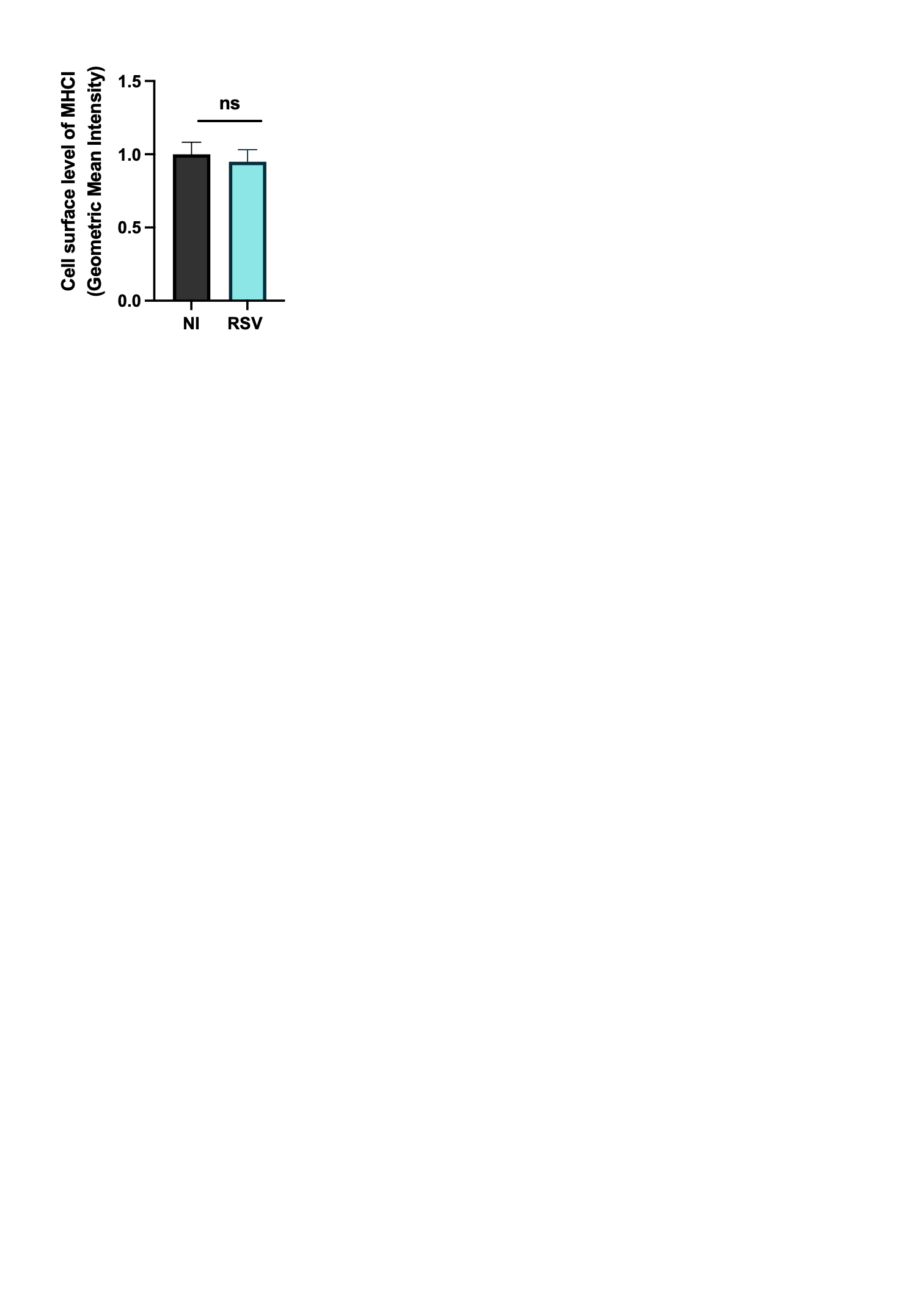

Supplement: S5 Fig — HEp–2 cells were infected or not with RSVmCherry wt. At 22h pi cells were fixed and stained with an antibody against MHCI (without permeabilization, dilution 1/100 [68]). The Geometric Mean Intensity (GMI) of MHCI at the surface of infected or not infected cells was determined by Flow Cytometry. Biological triplicates were performed for each condition. Data from 3 independent experiments. (TIF) [file ppat.1012687.s005.tif]

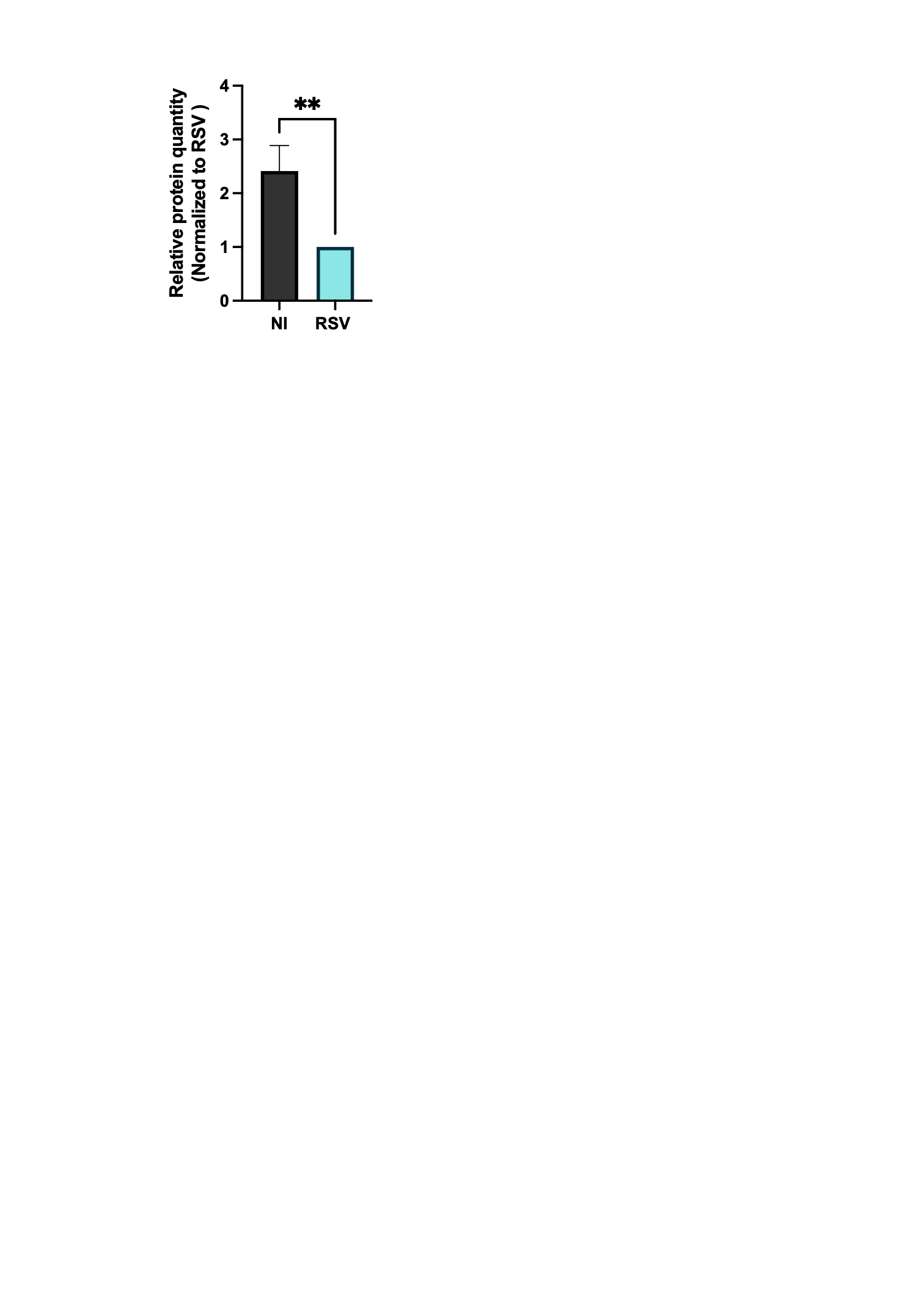

Supplement: S6 Fig — The relative quantity of BST2 protein was calculated using the Biorad Image Lab Software normalized to the protein quantity of the infected condition. Data from 3 independent experiments. (TIF) [file ppat.1012687.s006.tif]

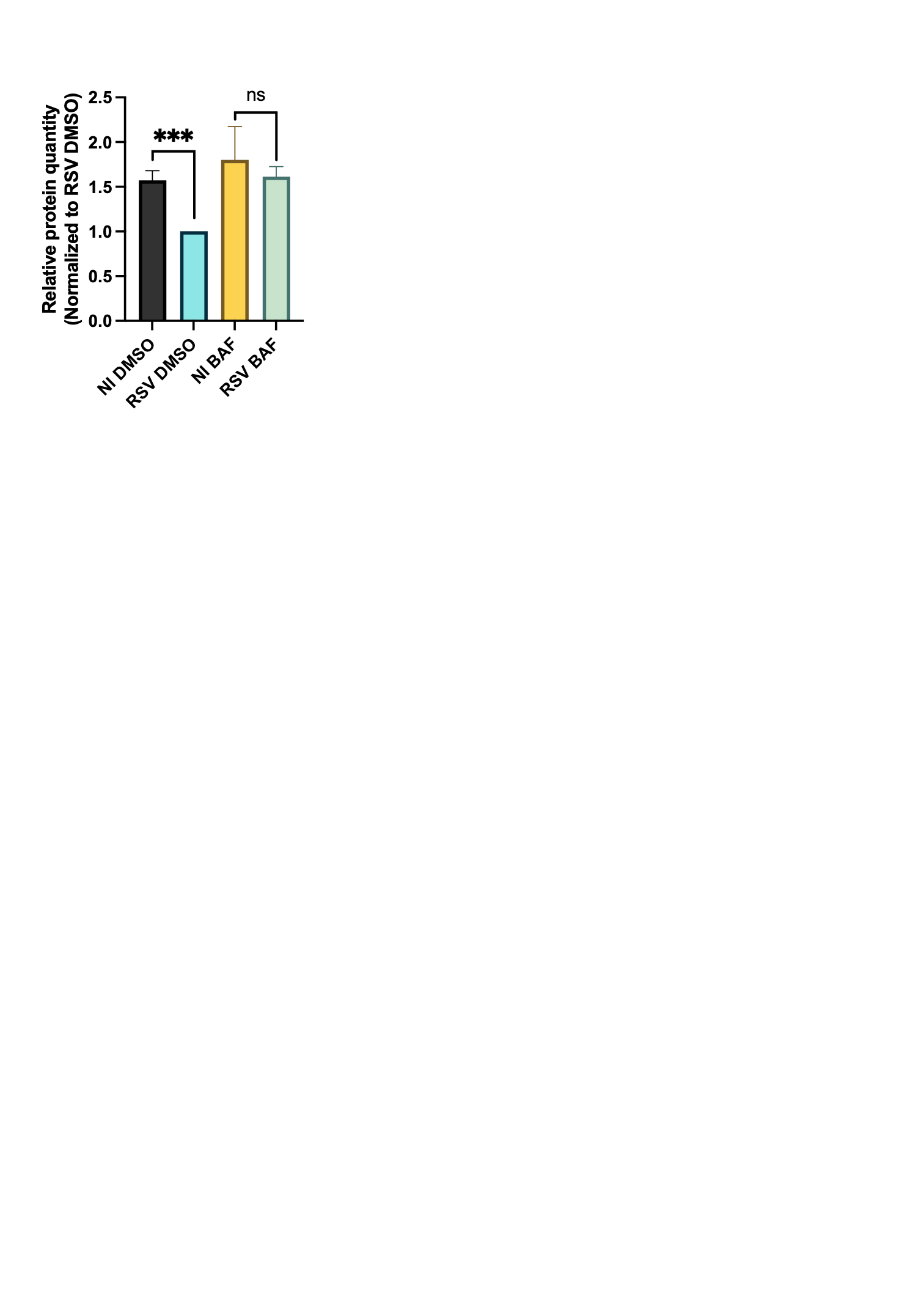

Supplement: S7 Fig — The relative quantity of BST2 protein was calculated using the Biorad Image Lab Software normalized to the protein quantity of the infected condition treated with the lysosomal degradation inhibitor Bafilomycin A1 or with DMSO. Data from 3 independent experiments. (TIF) [file ppat.1012687.s007.tif]

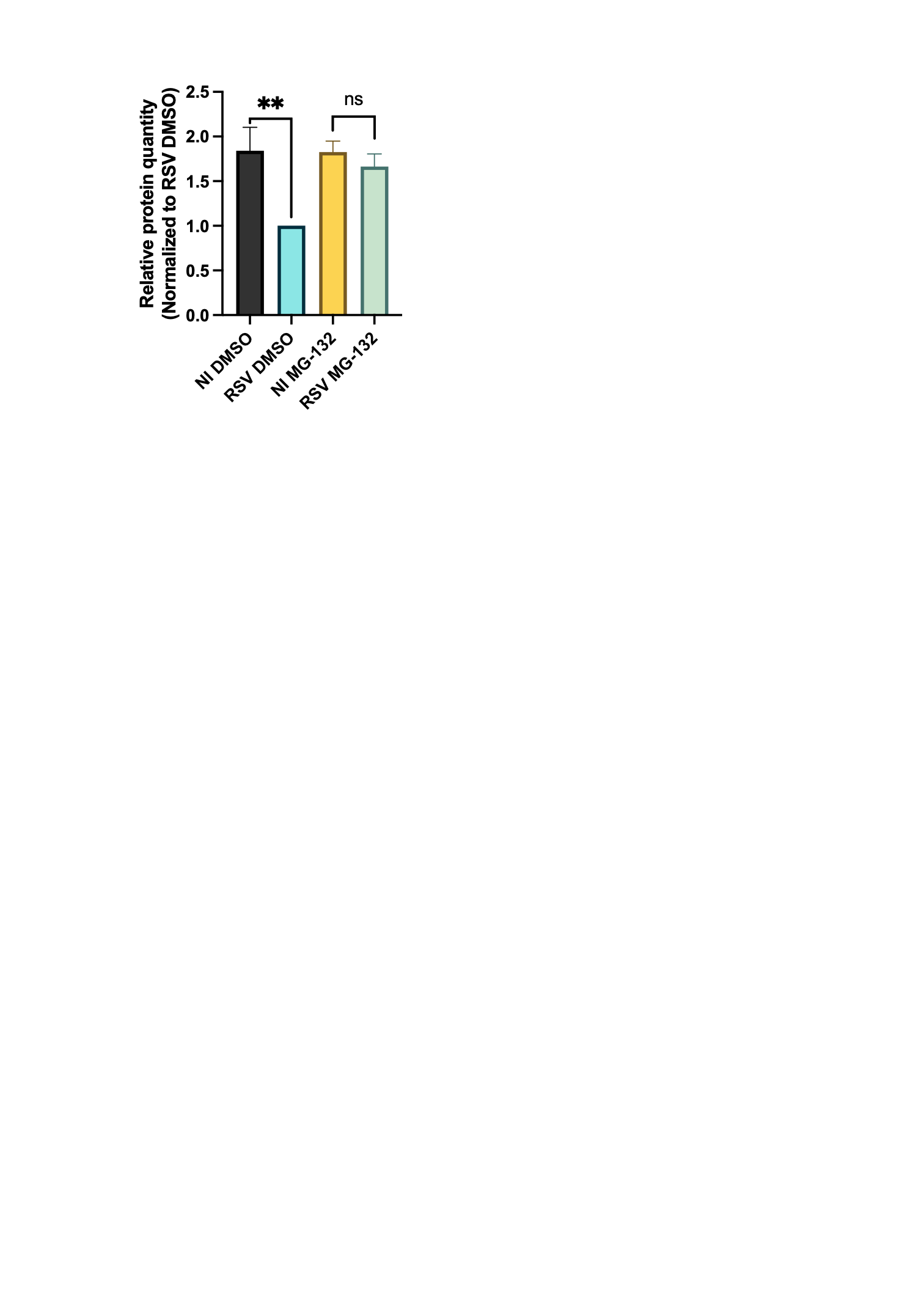

Supplement: S8 Fig — The relative quantity of BST2 protein was calculated using the Biorad Image Lab Software normalized to the protein quantity of the infected condition treated with the proteasomal degradation inhibitor MG-132 or with DMSO. Data from 3 independent experiments. (TIF) [file ppat.1012687.s008.tif]

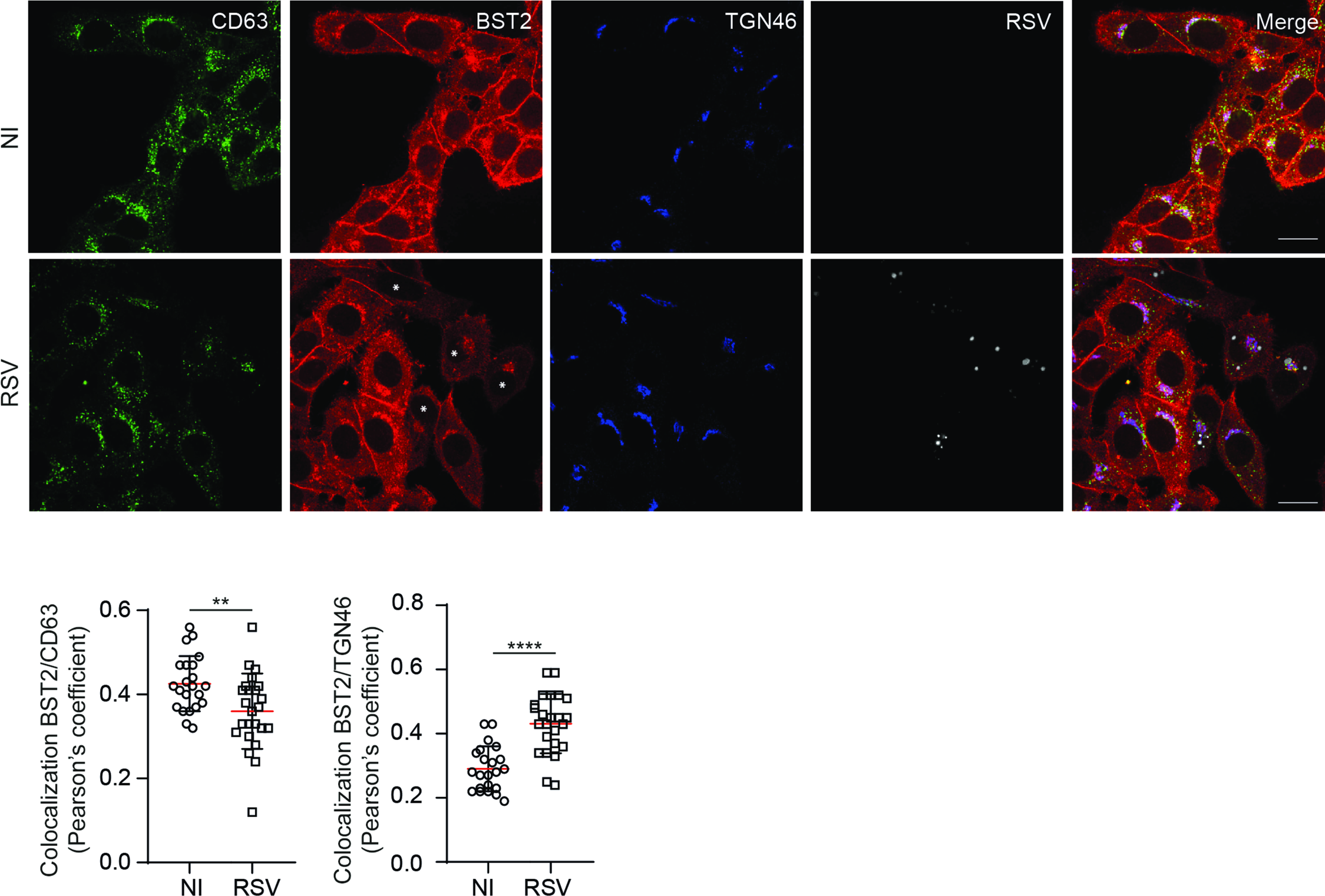

Supplement: S9 Fig — Confocal fluorescence microscopy of HEp-2 cells infected with RSV P-BFP at a MOI of 0,5 for 24hrs following total staining with mouse anti-CD63, rabbit anti-BST2 and sheep anti-TGN46 antibodies. Scale Bar: 20μM. Quantification of BST2 and CD63 or BST2 and TGN46 colocalization, Mean ± SD, n = 1 experiment, Pearson’s coefficient was measured in ∼20 cells per condition, and statistical analysis was done using Unpaired t test; **, P ≤ 0.01, ***, P ≤ 0.0001. (TIF) [file ppat.1012687.s009.tif]

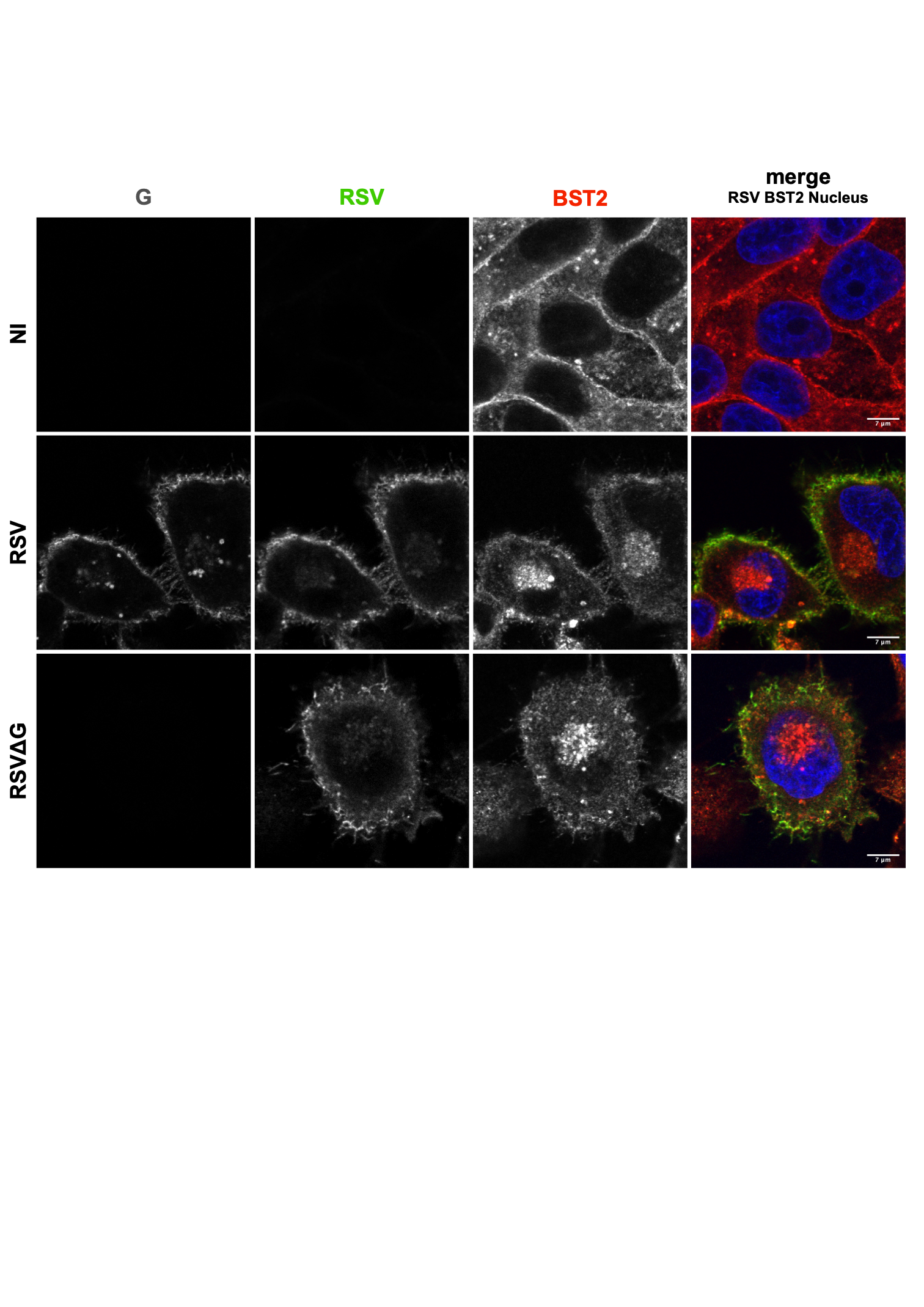

Supplement: S10 Fig — HEp-2 cells were infected or not with RSV and RSVΔG virus. At 24h p.i. cells were fixed and stained against BST2 (red in merge), G and RSV proteins (green in merge). Hoechst 33342 staining is shown in blue (merge). NI (not infected). Scale bar 7 μm. (TIF) [file ppat.1012687.s010.tif]

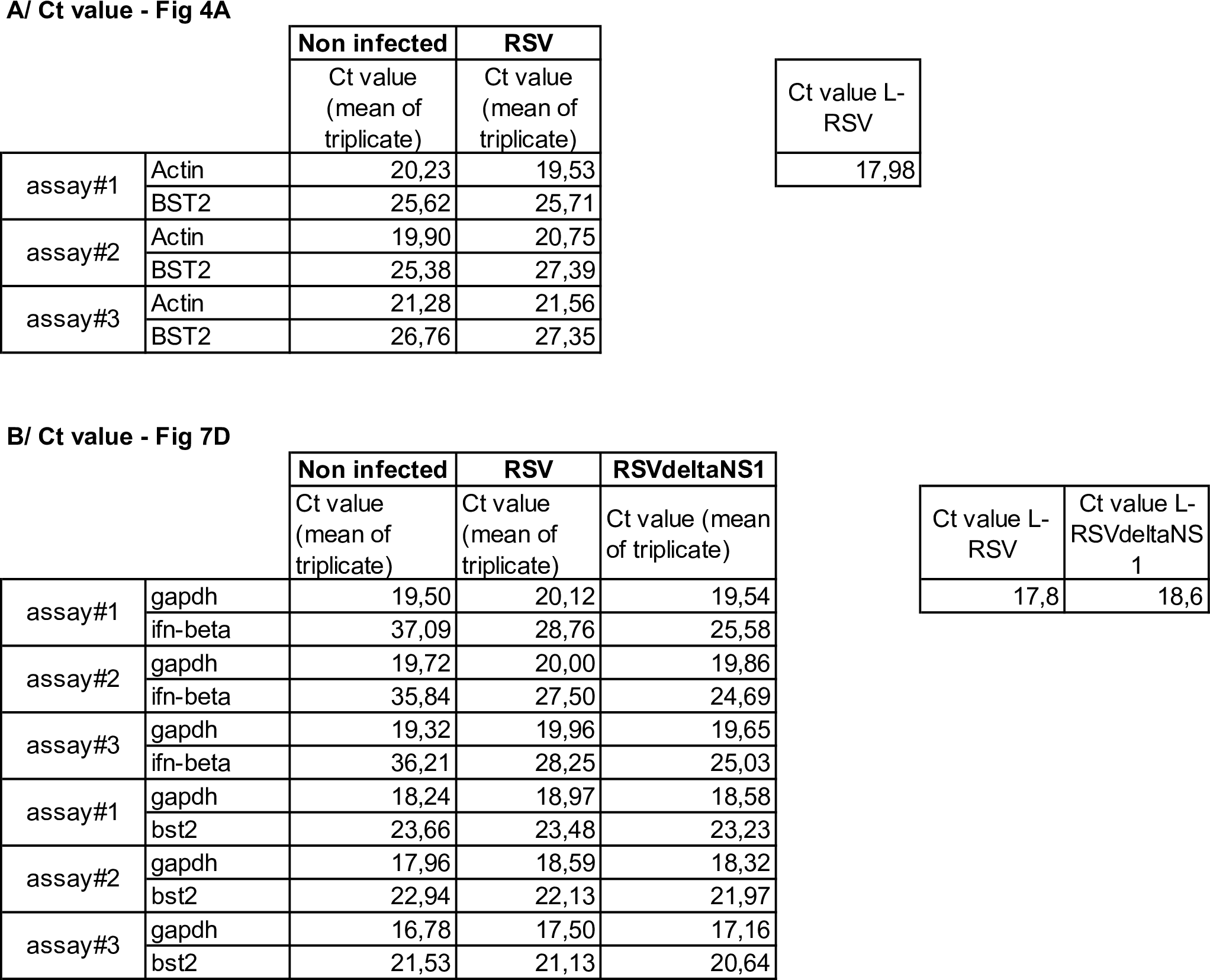

Supplement: S1 Table — Ct Value (mean of triplicate) for 3 independent experiments. (TIF) [file ppat.1012687.s011.tif]

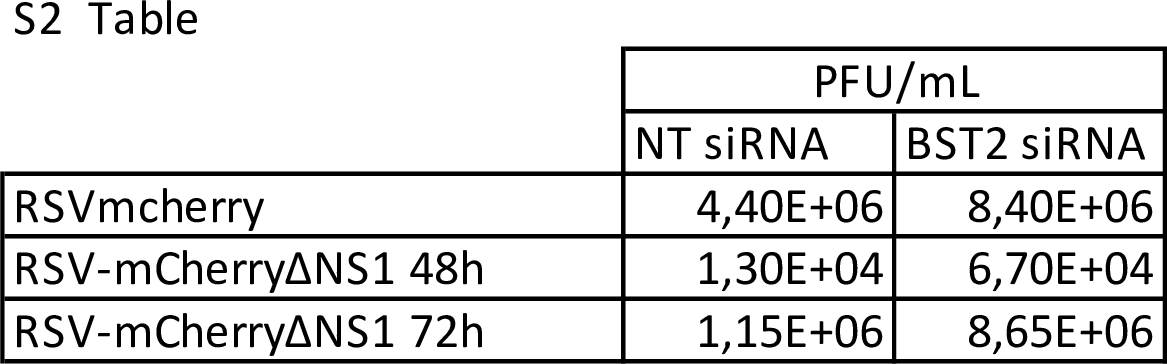

Supplement: S2 Table — (TIF) [file ppat.1012687.s012.tif]

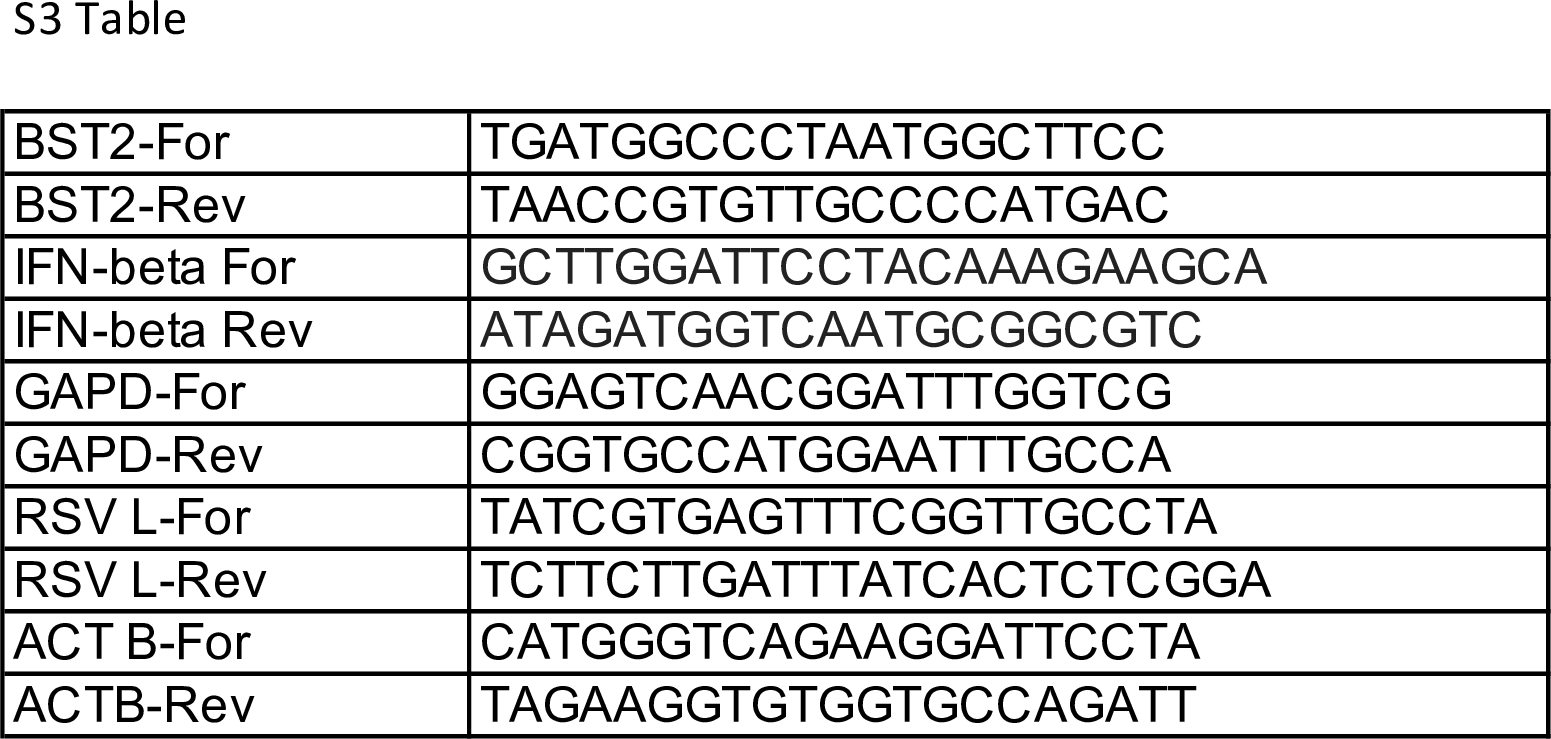

Supplement: S3 Table — (TIF) [file ppat.1012687.s013.tif]
