# Supplementary material for: Antagonism of BST2/Tetherin, a new restriction factor of respiratory syncytial virus, requires the viral NS1 protein
Source: PLoS Pathog. 2024 Nov 19;20(11):e1012687. doi: 10.1371/journal.ppat.1012687 (PMC11614281; doi:10.1371/journal.ppat.1012687)
Supplement: Attachment — Submitted filename: Response to reviewers.pdf [file ppat.1012687.s014.pdf]

Reviewer's Responses to Questions

Part I - Summary
Please use this section to discuss strengths/weaknesses of study, novelty/significance, general execution and scholarship.

Reviewer #1: In the current study the authors look at the impact of the anti-viral restriction factor BST2 on RSV infection in vitro. it is an interesting paper but more work is needed to confirm the mechanism that RSV evades the BST response and that it is a direct NS1 effect.

Reviewer #2: The manuscript titled "BST2/Tetherin, a new restriction factor of respiratory syncytial virus 1, is antagonized by the viral NS1 protein" is a research article describing the interaction of BST2/Tetherin in RSV yinfections in vitro. The authors also explored a potential evasive effect of RSV on BST2 induced by the viral protein NS1. While the authors presented convincing evidence of BST2's interaction with RSV, the demonstration of the antagonistic effect by NS1 requires further investigation. The paper has the potential to advance the knowledge of RSV biology. However, in its current form, the manuscript is too preliminary for acceptance. The reasons for rejection and suggestions to improve the manuscript quality are outlined below.

Reviewer #3: Marougka and colleagues investigated whether the cellular restriction factor tetherin/BST2 Is active against RSV. In brief, they show that siRNA-mediated knock-down of tetherin slightly increases RSV titers in a cell line at a late stage of infection. Further, an interaction of tetherin with components of the viral envelope is demonstrated and it is shown that RSV downregulates tetherin expression in a NS1 dependent fashion and that a delta NS1 virus is more sensitive to tetherin's antiviral activity than the WT virus. The findings are of interest to the field. However, important points remain open.

Part II – Major Issues: Key Experiments Required for Acceptance
Please use this section to detail the key new experiments or modifications of existing experiments that should be <u>absolutely</u> required to validate study conclusions.

Generally, there should be no more than 3 such required experiments or major modifications for a "Major Revision" recommendation. If more than 3 experiments are necessary to validate the study conclusions, then you are encouraged to recommend "Reject".

Reviewer #1: Major comments:
1. How do you separate out the broader anti-IFN role of NS1 from the specific anti-BST2 role? NS1 antagonises IFN, which would increase BST2, in its absence you will get more of both.

We thank the reviewer for this comment. Indeed, this is a key point of this article. First of all, as it is shown in Fig 3D, BST2 mRNA levels are not modulated (or are slightly increased) during RSV infection in HEp-2 cells. It must be specified that the normal mRNA level of BST2 in HEp-2 cells is already high (to clarify this point we had a supplementary table 1 with the Ct value of the RT-qPCR, as it is request by the reviewer #2). Moreover, to demonstrate that NS1 does not affect BST2 transcription (due to its broader anti-IFN role), we performed RT-qPCR experiments comparing BST2 transcripts during infection by RSV-mCherry or RSV-mCherry ΔNS1. We do not observe significant differences between both conditions (Fig 7D), whereas, IFNβ, a known target of RSV nonstructural proteins (Ren et al. J Gen Virol 2011, Ban et al. Viruses 2018), is significantly more expressed in RSV-mCherry ΔNS1 infected cells than RSV infected cells. In conclusion, in HEp-2 cells, the broader anti-IFN role of NS1 does not target BST2 and we can conclude that the post transcriptional modulation of BST2 by NS1 is specific.

We modified the text lines 346 to 355 (results) and 457 to 459 (discussion) and add Fig 7D.

2. Need to repeat the studies with an NS2 knockout virus and change line 303 – there is NS2 protein in the pull down, it might have a role.

We showed a pull-down assay demonstrating that NS1 was clearly immunoprecipitated together with BST2 and that NS2 alone was slightly immunoprecipitated with BST2. As NS2 immunoprecipitation was not observed when NS proteins were expressed together (as in the viral context), we then focused on the role of NS1.

As recommended by the reviewer, to explore the hypothesis of NS2 involvement, we should have performed a similar experiment as in figure 7C with RSVmCherry-ΔNS1-2 and RSVmCherry-ΔNS2 viruses. Unfortunately, we

are not able to rescue the RSVmCherry-ΔNS2 virus. Nevertheless, we performed the experiment with the RSVmCherry-ΔNS1-2 (figure below). Results of experiments showed that RSV-mCherry ΔNS1-2 is not more sensitive to BST2 restriction compared to RSV-mCherry ΔNS1, meaning that if NS2 is involved in the restriction, it is not the most important element of the restriction and for that we did not explore further in this paper. Further experiments would have to be undertaken to address this question but elucidating the role of NS2 is beyond the scope of this manuscript.

We changed the line 326 (303 in the previous version) and moderated our comments (line 483) in the discussion.

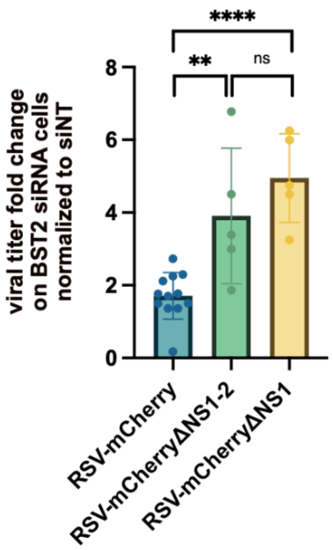

HEp–2 cells were transfected with control siRNA (NT) or siRNA targeting BST2. Forty-eight hours post transfection, cells were infected with RSV-mCherry or RSV-mCherryΔNS1 at low MOI (0.01). At 48h post infection, viral titers were measured by plaque titration assay. Viral titer fold changes between siRNA BST2 and NT HEp-2 cells were represented for the 3 viruses. Non-parametric unpaired *t*-tests were performed (ns not significant, ** $p<0.01$, **** $p<0.0001$).

Reviewer #2: It is widely accepted that Interferon-induced genes (ISGs) are not highly expressed in unstimulated cells, with exceptions noted in certain types of tumors characterized by elevated ISG expression. During the viral entry process, viruses can activate Pattern Recognition Receptors (PRRs). This activation triggers a cascade culminating in the expression and secretion of type I interferons (IFN). The paracrine and autocrine activation of Interferon receptors activates the STAT family proteins resulting in the expression of ISGs. The authors affirm that this manuscript seeks to investigate BST2's interaction with RSV under more physiological conditions than currently available in the literature. However, despite their objective to study physiological conditions, the chosen cell line for this investigation presents numerous inconsistencies and is unlikely to serve as an appropriate model. According to the ATCC website, the "HEp-2 cell line contains HeLa marker chromosomes and was derived via HeLa contamination(...)." (https://www.atcc.org/products/ccl-23). The uncertainty surrounding the cell lineage and the presence of high basal expression of BST2 create challenges in interpreting the results. I recommend that the authors redirect their efforts toward BCi-NS1.1 cells. If the authors decide to keep Hep-2 cells (possibly HeLa cells) they should authenticate their Hep-2 cell line avoiding future inconsistencies in the literature. The authors should also verify the effect of IFN stimulation and downstream effectors activation in their models, checking if the expression of BST2 in Hep-2 cells resembles the one in BCi-NS1.1 cells. Beyond the cell-related concerns regarding this manuscript, Marougka et al. did not attempt to elucidate the mechanisms underlying BST2 downregulation by NS1 as well. For instance, the only evidence provided regarding mechanisms relies on bafilomycin and MG132-treated cells. They should corroborate their drug-based assay by measuring mono-ubiquitination and poly-ubiquitination of BST2, as well as performing confocal experiments aiming to colocalize BST2 and acidic compartments or proteasome components.

The HEp-2 cell line is widely used in RSV research and can be very frequently found in the literature (Sake et al. Antimicrobial agents and Chemotherapy 2022, Ban et al. Viruses 2018). While BCi-NS1.1 cells are a very interesting model, that allowed us to observe that infected cells counteract BST2 expression in primary cells, its use is very limited. For example, they cannot be easily transfected by BST2 siRNA or by expression vectors of BST2, NS1 or NS2. In addition, they are cultured in small inserts that are limiting for experiments such as immunoprecipitations. It should also be taken into account that differentiation and infection experiments with BCi-NS1.1 last 3 to 5 weeks thus slowing down considerably the progress of the project.

In our initial manuscript our insight into the mechanism underlying BST2 downregulation by NS1 was limited to the use of degradation inhibitors. We gladly heeded the advice of the reviewers and performed additional experiments to better understand the mechanism. Specifically, Blotting with anti-ubiquitin antibodies BST2 immunoprecipitation revealed higher amounts of ubiquitinated forms of BST2 in RSV infected cells that in the non-infected condition (Fig 4E), suggesting that RSV infection increases BST2 ubiquitination.

Additionally, new immunofluorescence experiments (as suggested by the reviewer) were performed with CD63 antibodies, together with TGN46 and showcased that upon infection, BST2 is downregulated and mainly present in TGN46 positive compartments while less present in CD63 compartments. A possible explanation could be that an increased ubiquitination of BST2 during infection accelerated its degradation.

These data were introduced in Fig 4E and supplementary S8 Fig and lines 235 to 244.

Additional comments and suggestions:
1. Fig 1B. The authors suggest that BST2 depletion improves viral production. Fig 1B shows a multi-step replication curve of RSV. The authors called the difference found modest but highly reproducible. RSV can present a very long replication cycle and 48 hours may not reflect a truly multistep curve. I advise the authors to define the time required for a one-cycle curve in the cell lineage they are using and perform a multicycle curve based on the one-cycle curve timeframe.

In our experience (illustration on the figure below), RSV wild type replication reaches the “plateau” stage after 18-20 hours of infection. For that reason, we believe that 48h post infection is sufficient to observe multicycle kinetics.

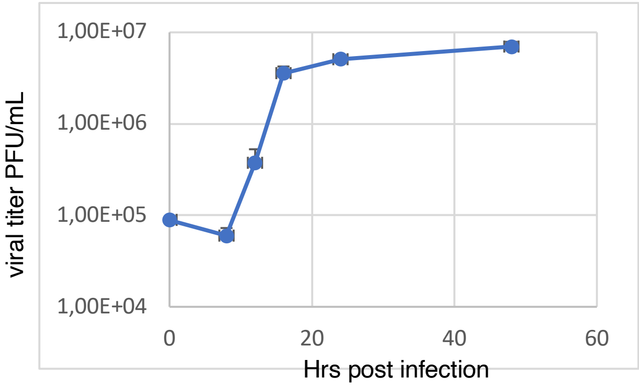

HEp-2 cells were infected at an MOI of 1 at 37°C. Viruses were harvested at the indicated times p.i. and titrated by plaque assays on HEp-2 cells. Mean of 3 independent experiments

2. In Fig 3B, the authors quantified the amount of BST2 in the plasma membrane, however, no plasma membrane marker was used. The use of phalloidin to reconstruct the cellular plasma membrane may not generate an accurate location of the plasma membrane. If the authors think that there are no better options, the authors should describe better how the plasma membrane reconstruction and BST2 localization/quantification were performed. Additionally, the authors should clarify why the total amount of BST2 is based on the presence of BST2 in TGN (along with the plasma membrane) instead of the total amount of BST2 present in the whole cell.

The entirety of each cell was captured by performing a Z-stack acquisition (0,35 µm step). As most of the BST2 signal was concentrated at the Trans-Golgi-Network and the cell surface, we decided to measure the amount of BST2 at these two parts of the cell and add them together as an approximation of the total amount of BST2 present in the cell. GM130 was chosen as the Trans-Golgi-Network BST2 marker. Unfortunately, Phalloidin was the only compatible tool we had in the laboratory when the experiment was set up.

3. In lines 222-224, the authors affirm that "We showed in Fig 3D that BST2 mRNA level was not significantly modified in infected cells compared to non-infected cells, suggesting that the regulation of the amount of BST2 doesn’t take place at the transcriptional level." The use of relative quantification in their analysis avoids the detection of global expression down-regulation, which is common for many virus families. Caution should be exercised in this statement, and some other method should corroborate such conclusion. Additionally, providing the primer sequences and the reference gene used for the relative quantification would enhance the transparency and reproducibility of the study.

We thank the reviewer for this comment and clarify now our experiment. Primer sequences and Ct value of the experiment are available on S1 and S3 Tables respectively. The normal mRNA level of BST2 in Hep2 cells is already high and is not modulated (or are slightly increased) during RSV infection.

4. Comparing Hep-2 cells (possibly HeLa cells) and BCi NS1.1 cells is problematic. The former expresses a high level of ISGs in the absence of IFN activation, while the latter appears to depend on virus infection and likely IFN stimulus. These are entirely different biological models and should not be directly compared unless experimental evidence proves otherwise.

We did not compare directly HEp-2 and BCi NS1.1 cells. In contrast, it is interesting to observe that, in cells that express high level of BST2 in the absence of IFN activation and in others that require the stimulus of viral infection, viral antagonists potentially employ similar strategies to overcome restriction by BST2. However, elucidation of precise mechanisms taking place in BCi NS1.1 cells is beyond the scope of this manuscript.

We modified the text to clarify our conclusion (line 441)

5. Marougka et al also failed to quantify the expression of BST2 mRNA in the experiments with RSVΔNS1, in the assays using BCi NS1.1 cells, and in the experiments using plasmid transfections. The quantification of BST2 mRNA should be part of all experiments comparing BST2 variations.

We thank the reviewer for this comment which allowed us to demonstrate that NS1 does not affect BST2 transcription (due to its broader anti-IFN role). Indeed, we performed qRT-PCR experiments comparing BST2 transcripts during infection by RSV-mCherry or RSV-mCherry ΔNS1. We did not observe significant differences between both conditions (Figure 7D), whereas, IFNβ, a known target of RSV nonstructural proteins (Ren et al. J Gen Virol 2011, Ban et al. Viruses 2018), is significantly more expressed in RSV-mCherry ΔNS1 infected cells than RSV infected cells. In conclusion, in HEp-2 cells, the broader anti-IFN role of NS1 does not impact BST2 mRNA expression and we can conclude that the post transcriptional modulation of BST2 by NS1 is specific.

We modified the text lines 346 to 355 (results) and 457 to 459 (discussion) and add Fig 7D.

Unfortunately, as mentioned above further experiments in BCi NS1.1 cells are not feasible.

6. Bafilomycin inhibits endosome acidification, and MG132 inhibits proteasome-mediated degradation. Although inhibitory drugs can serve as initial evidence for biological phenomena, the effect is not specific. Drugs frequently affect multiple processes inside the cells and a more specific approach should be used to corroborate the data using drugs.

7. The conclusion that BST2 degradation upon infection is dependent on the endolysosomal pathway and ubiquitin is not supported by the manuscript data. MG132 inhibits proteasome-mediated degradation but does not directly influence ubiquitination.

6-7 As mentioned above, in our initial manuscript, our insight into the mechanism underlying BST2 downregulation by NS1 was limited to the use of degradation inhibitors. We gladly heeded the advice of the reviewers and performed additional experiments to better understand the mechanism. Specifically, Blotting with anti-ubiquitin antibodies BST2 immunoprecipitation revealed higher amounts of ubiquitinated forms of BST2 in RSV infected cells that in the non-infected condition (Fig 4E), suggesting that RSV infection increases BST2 ubiquitination.

Additionally, new immunofluorescence experiments (as suggested by the reviewer) were performed with CD63 antibodies, together with TGN46 and showcased that upon infection, BST2 is downregulated and mainly present in TGN46 positive compartments while less present in CD63 compartments. A possible explanation could be that an increased ubiquitination of BST2 during infection accelerated its degradation.

These data were introduced in Fig 4E and supplementary S8 Fig and lines 235 to 244.

8. The authors' statement "an RSVΔNS1 virus is more sensitive to BST2 restriction than the wild-type virus because it fails to decrease BST2 cell surface expression" is supported by comparing Fig 6A's bottom panel and middle

panel. However, the bottom panel in Fig 6A resembles the same pattern observed in the bottom panel in Fig 3B. The authors should discuss why RSV infection in Fig 3B resembles RSVΔNS1 infection in Fig 6A.

We agree with the referee, first set of images was not well representative of our results, we modified the bottom panel of Fig 7A (previously 6A).

Reviewer #3: The effect of tetherin knock-down on RSV infection is minor. Confirmation of antiviral activity is needed under conditions of overexpression. Thus, infection of cell lines overexpressing tetherin relative to control cell lines must be analyzed.

We thank the reviewer for allowing us to carry out this experiment. As transient transfection prevents RSV infection, we used episomal cell lines overexpressing BST2 (used in figures 2B and 2C), to study the effect of BST2 overexpression on RSV infection. The cell lines were infected with a recombinant RSV virus expressing luciferase (RSV-Luc) at low MOI (0,01), after 48h total lysates were analysed by a luciferase assay. The choice of performing the experiment with the RSV-Luc virus rather than the one shown in Figure 1 is due to a lower susceptibility of infection of the episomal cell lines. As shown on Supplementary data S2, luciferase expression was 3-fold less in cells overexpressing BST2. These results complement the knock down experiments and confirm that BST2 acts as a restriction factor of RSV infection. We added a supplementary Fig S2 and modified the text from lines 140 to 145.

Further, it would be more convincing to show the average of several independent experiments in figure 1B as compared to the results of a representative experiment.

Figure 1B shows raw data representing viral particle production. Depending on the experiment, we obtain values with different orders of magnitude, making it difficult to pool. To pool 3 independent experiments, we measured the fold change between BST2 siRNA and control siRNA at 36h and 48h (as represented on the Fig 7D). Results showed a significant fold change at 48h but not at 36h (figure below).

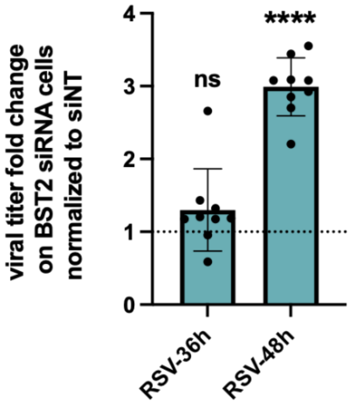

HEp–2 cells were transfected with control siRNA (siNT) or siRNA targeting BST2. Forty-eight hours post transfection, cells were infected with RSV at low MOI (0.01). At 36h and 48h post infection, viral titers were measured by plaque titration assay. Viral titer fold changes between siRNA BST2 and NT HEp-2 cells were represented. One sample t-test is no significantly different from 1 at 36h but significantly at 48h (**** *p<0,*0001)

It is important to investigate whether NS1 alone like Vpu is able to antagonize tetherin. This question can be addressed in a straightforward manner by analyzing whether NS1 expression rescues release of retroviral particles from inhibition by tetherin.

As asked by reviewer #3, we investigated the ability of NS1 to rescue the release of retroviral particles from inhibition by BST2/Tetherin. For that, we analysed the release of a mutated HIV-1 virus that do not express Vpu, the HIV antagonist of BST2/Tetherin, in cells transfected with Vpu-deleted HIV-1 provirus (HIV-1 Udel) and either NS1 or NS2 or NS1 + NS2. As shown in the figure below, neither NS1 nor NS2 nor NS1 in combination with NS2 are able to antagonize BST2 inhibitory effect on HIV release, indicating that NS1 alone or in complex with NS2 are not sufficient to antagonist BST2 antiviral effect on HIV-1 release. This suggests that the mechanism by which NS1 antagonist BST2 restriction in the course of RSV infection does not mimic those developed by HIV-1 Vpu to counteract BST2 restriction on HIV release. It can also postulate that NS1 needs additional viral proteins to counteract BST2 restriction. This additional factor is not NS2 since addition of NS2 in combination with NS1 in our experiment does not rescue viral release. Additional experiments, beyond the scope of this manuscript, would be set up to explore the contribution of other RSV proteins acting in concert with NS1 to antagonist BST2, such as F, N proteins that we found associated to BST2 in RSV infected cells (s) or the SH proteins, a small transmembrane protein resembling to viroporine like HIV-1 Vpu.

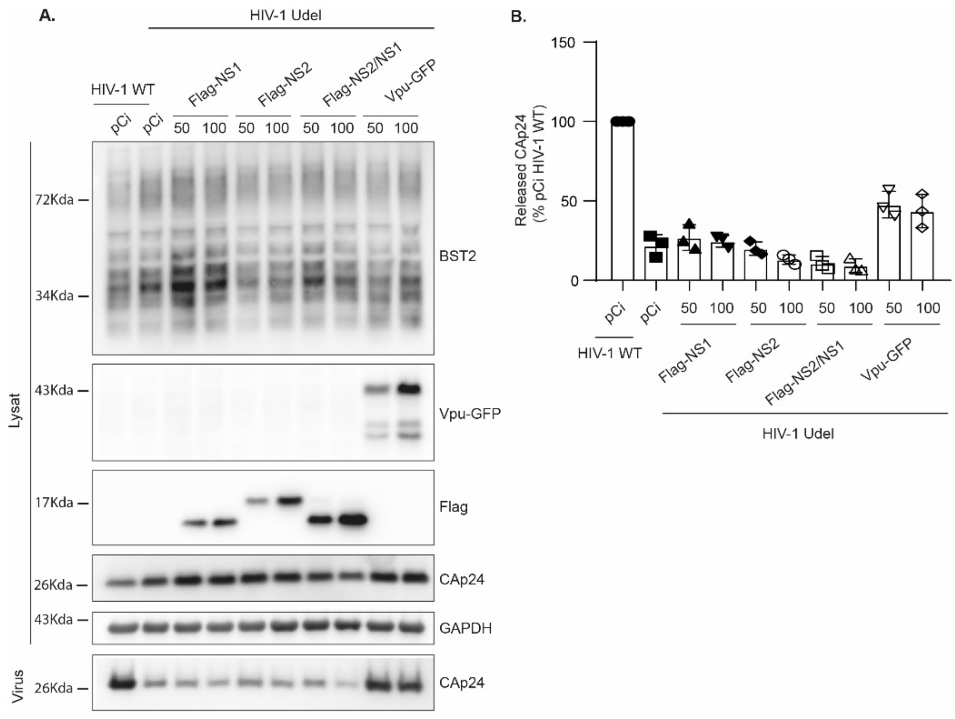

HeLa cells were co-transfected with provirus HIV-1 WT or Vpu-deleted (Udel) in combination with empty vector (pCi) or expression vector encoding Flag-NS1, Flag-NS2 or Vpu-GFP. 48hrs post-transfection cell lysates and pelleted supernatants were analysed for HIV-1 CAp24 by western blot (A) and ELISA (B). Cell lysates were also analysed for Flag-NS1, Flag-NS2, Vpu-GFP and GAPDH by western blotting. Western blot are representative of at least three independent experiments. n=3 experiments.

Figure 6C: It is important to state the viral titers in the figure legend. If delta NS1 virus grew to much lower titers as compared to WT virus then the data are not very telling.

We thank the reviewer for this observation. As indicated in the first version of the manuscript, the RSV-mCherry ΔNS1 virus has a reduced fitness compare to RSV-mCherry virus (we now added the average of the viral titers in the supplementary table S2). Then, to compare the restriction "level" of BST2 between these two viruses, we compared the difference between ctrl siRNA and BST2 siRNA for each virus. In the new version of the manuscript, to confirm that RSV-mCherry ΔNS1 is much more sensitive to BST2 restriction, we repeated the experiment and included an additional time point at 72h pi for RSV-mCherry ΔNS1 virus. In accordance to our previous results, the difference in fold-change between knock down cells infected with RSV-mCherry or RSV-mCherry ΔNS1 was not only maintained but increased even more.

Figure 7 (previously Fig 6) was modified as mentioned above.

Part III – Minor Issues: Editorial and Data Presentation Modifications
Please use this section for editorial suggestions as well as relatively minor modifications of existing data that would enhance clarity.

Reviewer #1: Minor comments:
1. Why not take the in vitro assay out longer in Fig 1B? The effect only apparent after 48 hrs, can you look at 72?

In our experience (illustration on the figure below), RSV wild type replication reaches the "plateau" stage after 18-20 hours of infection. For that reason, we believe that 48h post infection is sufficient to observe multicycle kinetics.

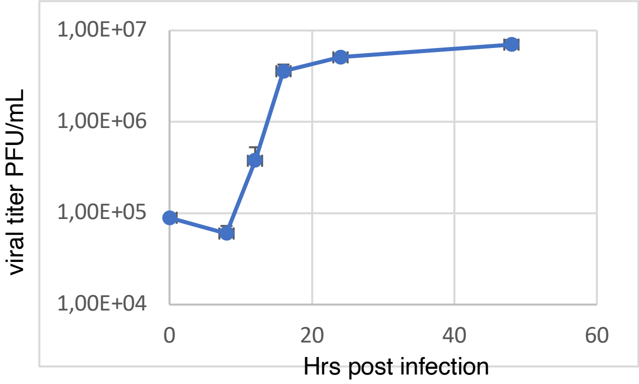

HEp-2 cells were infected at an MOI of 1 at 37°C. Viruses were harvested at the indicated times p.i. and titrated by plaque assays on HEp-2 cells. Mean of 3 independent experiments

2. In 2A: the F blot, what is the higher band that is present in all the lanes?

The higher band present in all the lanes is a non-specific signal caused by the anti-F antibody.

3. Why is there BST2 in the HeLa NI images but not the primary cells? Does that affect interpretation?

Indeed, the two cell lines differ in the expression of BST2 in non-infected cultures. In HEp-2 (Hela derivative), a cell line widely used for RSV research, BST2 is already expressed in NI cells. Upon infection, BST2 mRNA level is not significantly changed as shown on Fig 4A and our data show that RSV modulates BST2 post-translationally. However, in the differentiated cells, BST2 is not expressed in NI cells, infection induced BST2 (as an ISG gene), but in the specific cells replicating the virus, we do not observe BST2 signal. It is interesting to observe that, in HEp-2 cells expressing high level of BST2 in the absence of IFN activation and Bci NS1.1 cells requiring stimulus of viral infection for BST2 expression, viral antagonists potentially employ similar strategies to overcome restriction by BST2. However, elucidation of precise mechanisms taking place in BCi NS1.1 cells is not the subject of this manuscript (the analysis in the primary cells requires additional tools that are very difficult to implement)

Reviewer #2: Minor issues

1) Fig 4, if the monolayer was infected in a high MOI, why just a small fraction of cells is infected? The MOI is defined using a theoretical value (obtained from titrations performed on Hep2 cells), but it is not necessarily adapted to BCi NS1.1 cells, which are less sensitive to infection. These cells have previously been shown to be susceptible to RSV infection (Sake et al.). This point has been clarified line 288

2) Flow cytometry histograms should be displayed in the figures.

A replicate from the dataset represented in Figure 3A is displayed below. Non infected cells are depicted in grey and infected cells in blue. As expected, the peak of the infected cells' histogram is shifted towards the left as RSV infected cells display less (30%) BST2 at their surface. We agree that the decrease is low but highly reproductible, and we would prefer to maintain the representation of our data as columns in Figure 3A.

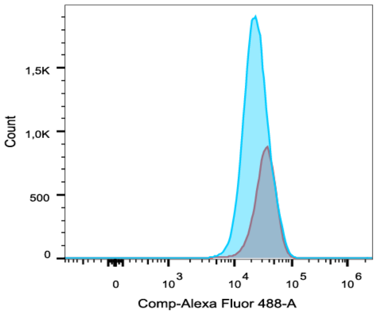

Y axis : cell count

X axis : BST2 signal

3) RSVΔNS1 should be added to Fig 1B. Fig1B is showing a wild type RSV virus and not the recombinant RSV-mcherry virus. We compared RSV-mCherry ΔNS1 virus and RSV-mcherry WT later in the manuscript (Figure 7)

4) Fig 1B legend: What does “Representative data of 3 independent experiments are shown” mean? Does it mean that only data from one experiment was used in the graph? If that is the case, the authors should add the other experiments to the graph as well.

Figure 1B shows raw data representing viral particle production. Depending of the experiment, we obtain values with different orders of magnitude, making it difficult to pool. To pool 3 independent experiments, we measured the fold change between BST2 siRNA and control siRNA at 36h and 48h (as represented on the figures 7D). Results showed a significant fold change at 48h but not at 36h (figure below).

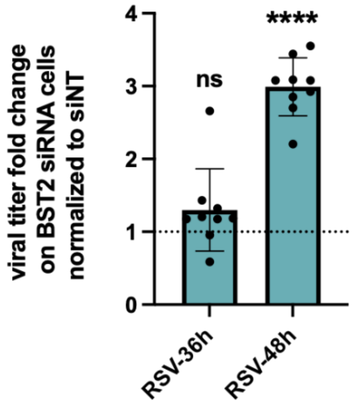

HEp–2 cells were transfected with control siRNA (siNT) or siRNA targeting BST2. Forty-eight hours post transfection, cells were infected with RSV at low MOI (0.01). At 36h and 48h post infection, viral titers were measured by plaque titration assay. Viral titer fold changes between siRNA BST2 and NT HEp-2 cells were represented. One sample t-test is no significantly different from 1 at 36h but significantly at 48h (**** *p<0,*0001)

5) In Line 219, the authors should add the Figure number to the flow cytometry results they mentioned.

Figure number of the cytometry results (Fig 3A) has been added to the new version of the manuscript.

6) In Fig 6A, cells in the bottom panel seem smaller than the middle and top panels. Could the authors verify if the magnification used is the same?

We thank the reviewer for the observation. The bottom panel has been corrected.

7) Lines 388-389: the authors mentioned that BCi-NS1.1 expression of BST2 is dependent on interferon secretion. Interferon detection was not performed in any of the experiments described in this manuscript.

Interferon detection was indeed not performed in this primary cells. We modified the text (line 291) to avoid overstating our results. However, a recent study showed that in human small airway epithelial cell, BST2 belongs to the core of IFN stimulated genes activated under RSV infection (Frontier in immunology, March 2024)

8) The authors should disclose enough information in the Material and Methods session to allow the reproducibility of the study. Information like antibody dilutions, primer sequences, confocal settings, and details in the image analysis are missing.

Requested informations were added to the revised manuscript.

9) Line 521-527. Is the BST2 quantification performed per z-stack? A better description of the image analysis should be added to the manuscript. The quantification was indeed performed per z-stack. Further details were added to the manuscript (lines 589 -590)

Reviewer #3: It is a significant weakness of the study that the impact of tetherin on RSV spread in primary lung cells and the contribution of tetherin to inhibition of RSV infection by IFN were not analyzed. This should be clearly stated in the discussion section.

While BCi-NS1.1 are a very interesting model, that allowed us to observe that infected cells counteract BST2 expression, its use is very limited. For example, currently they cannot be transfected by BST2 siRNA or by expression plasmids. In addition, they are cultured in small inserts that are limiting for experiments such us immunoprecipitations. Consequently, elucidation of precise mechanisms taking place in BCi NS1.1 cells is beyond the scope of this manuscript.

This point is now clearly stated in the discussion (line 474)
