# Supplementary material for: Antagonism of BST2/Tetherin, a new restriction factor of respiratory syncytial virus, requires the viral NS1 protein
Source: PLoS Pathog. 2024 Nov 19;20(11):e1012687. doi: 10.1371/journal.ppat.1012687 (PMC11614281; doi:10.1371/journal.ppat.1012687)
Supplement: Attachment — Submitted filename: Reviewer response.pdf [file ppat.1012687.s015.pdf]

Reviewer's Responses to Questions

Part I - Summary
Please use this section to discuss strengths/weaknesses of study, novelty/significance, general execution and scholarship.

Reviewer #1: The authors have addressed my concerns

Reviewer #2: The manuscript titled "BST2/Tetherin, a New Restriction Factor of Respiratory Syncytial Virus 1, is Antagonized by the Viral NS1 Protein" describes the interaction of BST2/Tetherin in RSV infections in vitro. In my initial review, I recommended the rejection of the paper due to its preliminary nature. While the revised version shows significant improvement, it remains fundamentally preliminary. Therefore, my recommendation remains rejection.

some of Marougka et al.'s conclusions appear somewhat rushed. For instance, they demonstrated that the ubiquitination of BST-2 increases in RSV-infected cells. This is interesting data; however, they fail to clarify how they differentiate between polyubiquitination (a signal for proteasome degradation) and monoubiquitination (a signal for endosomal degradation).

My comments and suggestions are outlined below.

Reviewer #3: The authors have assessed the effect of exogenous tetherin on RSV infection and now present the average of several experiments assessing the effect of tetherin on relative production of viral particles. This is appreciated and makes the manuscript stronger. However, two important points remain insufficiently addressed:

Part II – Major Issues: Key Experiments Required for Acceptance
Please use this section to detail the key new experiments or modifications of existing experiments that should be absolutely required to validate study conclusions.

Generally, there should be no more than 3 such required experiments or major modifications for a "Major Revision" recommendation. If more than 3 experiments are necessary to validate the study conclusions, then you are encouraged to recommend "Reject".

Reviewer #1: N/A thank you for softening the conclusion about NS1

Reviewer #2: In the current version, the authors have convincingly proved that 1) BST-2 is a restrictive factor for RSV infections; 2) BST-2 is present in the plasma membrane of uninfected cells; 3) when infected by RSV, the BST-2 migrates from the plasma membrane to acidic compartments; 4) the downregulation of BST-2 in Hep-2 cells infected by RSV is not at the transcriptional level; 5) RSV infection induces BST-2 ubiquitination in Hep-2 infected cells; 6) RSV NS1 protein immunoprecipitated together with BST-2 in Hep-2 infected cells; and 7) BST-2 in RSV ΔNS1 infected Hep-2 cells tends to stay at the plasma membrane when compared to wt RSV.

Some of Marougka et al.'s conclusions seem somewhat rushed. For instance, they demonstrated that the ubiquitination of BST-2 increases in RSV-infected cells and concluded that BST-2 is polyubiquitinated in these cells. While this is intriguing data, general ubiquitination staining cannot distinguish between polyubiquitination (a signal for proteasome degradation) and monoubiquitination (a signal for endosomal degradation). If the authors have differentiated mono and polyubiquitination in this manuscript is not clear how. They should clarify this issue by giving a better description in the material and method session.

We demonstrated figure 4E that ubiquitination of BST2 increases in RSV infected cells (lines 239-241). Based on these results, we concluded that “BST2 polyubiquitinylation and then degradation are

enhanced in infected HEp-2 cells" and in discussion section that "We also showed an increase of polyubiquitinated BST2 during infection". Reviewer #2 is right "general ubiquitination staining cannot distinguish between polyubiquitination and monoubiquitination".
Indeed, by performing Western blot analysis of ubiquitination status of deglycosylated BST2 with an anti-Ub FK2 antibody (that recognizes poly and mono-ubiquitinated conjugates), we are not able to distinguish mono- or poly- ubiquitinated BST2. Even if we clearly see on our Western blot (figure 4E) an accumulation of higher molecular weight forms of BST2 in RSV infected cells, suggesting an enhancement of BST2 polyubiquitination, our experimental approach does not allow to differentiate rigorously whether these higher molecular weight forms correspond to multi-monoubiquitinated or polyubiquitinated BST2.

To be more cautious, two conclusions were modified in the revised version of the manuscript, as follow:
-Line 248: Altogether, our data suggested that BST2 ubiquitinylation and then degradation are enhanced in infected HEp-2 cells, leading to a reduced exposure of BST2 at the plasma membrane.
-Line 449: We also showed an increase of ubiquitinated BST2 during infection and a decrease of BST2 presence in CD63 compartments compared to non-infected cells.
We also add in material and methods that anti-ubiquitin FK2 antibody recognizes either mono- and poly-ubiquitinylated conjugates (line 652).

BST-2 is described as a dimer, with the C-terminal GPI anchor trapping viral membranes at the plasma membrane. The authors demonstrated RSV trapped at the membrane in Figure 1, indicating that RSV is recognized by BST-2 in Hep-2 infected cells. If the authors' hypothesis is correct, RSV would escape being trapped by BST-2 at the plasma membrane, and RSV-ΔNS1 would accumulate at the plasma membrane. Figure S9 shows RSV colocalizing with BST-2 in areas close to the plasma membrane. In my opinion, this indicates that wt RSV cannot efficiently escape from BST-2. The authors did not show the same staining as Figure S9 for RSV-ΔNS1 for comparison purpose.

In Figure 2B and 2C, the high amount of BST2 observed at the membrane in association with viral particles is explained by the experimental conditions: In this experiment, as indicated in the main text (line 174), we analyzed the co-distribution of RSV and BST2 at the cell surface and for that we performed this experiment in HEP-2 cells overexpressing HA-BST2.

Focusing on Figure S9 (S10 in the new version), experiment was done in "normal" HEp-2 cells. We agree that BST2 and RSV particles are still present but at a lesser extent at the plasma membrane and in the membrane extension compared to the overexpressed condition (Fig 2B-2C). This signal in Figure S10 is in line with the results of Figures 3 and 7, where we still observed a small amount of BST2 at the membrane in RSV infected cells, probably because RSV cannot totally escape to BST2, as suggested by reviewer #2. To note, we modify the figure S10 which was confusing. Indeed, in the previous version of the manuscript, RSV proteins were shown in yellow and BST2 in red in the merge, which strengthen the co-localization effect. We now represent BST2 in red and RSV proteins in green in the merge (modification lines 934-37), which clarify the merge panel analysis. Moreover, we did not perform the same staining as Figure S9 (new figure S10) for RSV-ΔNS1 as mentioned by reviewer #2. But, in figure 7, we showed that BST2 is more present at the surface of cell infected with RSV-ΔNS1 than with WT RSV.

Finally, the reviewer #2 suggested that our results indicate that "wt RSV cannot efficiently escape from BST-2". Indeed, we cannot exclude that an additional factor traps RSV viral particle, as in all immortalized cell lines susceptible to RSV infection, it is known that RSV viral particles remain attached to cell membranes. We now added references to support this particular point (lines 137 and 442).

After being activated by viruses membranes, the complex BST-2-virus is endocytosed and the virus is inactivated at the acidic compartments. Part of the endocytosed BST-2 is recycled to the plasma membrane through the TGN. Therefore, the colocalization of BST-2 with TGN showed in Marougka et al does not necessarily mean that BST-2 is being targeted for destruction and may be the natural course of BST-2 after activated by viruses.

This is an interesting point. We observed a downregulation of BST-2 in Hep-2 cells infected by RSV on total protein extracts (Western blot; Figure 4B) while BST2 transcripts are unaffected (Figure 4A). Moreover, our data showed less amount of BST2 at the membrane of infected cells, and more BST2 accumulated in the TGN. Altogether, we could suggest that the recycling of BST2 could be altered in response of the infection, leading to BST2 degradation. Thanks to the reviewer, we now had this comment in the discussion (lines 451-454)

Viruses have evolved ways to evade BST-2 binding. The most studied strategy for BST-2 evasion is HIV's Vpu. Vpu seems to induce the degradation of BST-2 by proteasomes and inhibition of BST-2 recycling from TGN. The authors claim that a similar mechanism is observed for RSV NS1. In my opinion, this manuscript does not produce enough evidence to support their claim.

We apologize if our findings have been interpreted in this way. At the request of reviewer #3, we analysed whether NS1 expression is able to rescue release of retroviral particles from inhibition by BST2. Our results suggest that the mechanism by which NS1 participates to BST2 antagonism in the course of RSV infection does not mimic the strategy developed by HIV-1 Vpu to counteract BST2 restriction on HIV release. In this new version, the figure corresponding to these results has been added (Figure 8) and text modified (lines 359-365 in the results section and lines 507-509 in the discussion section).

Other major questions are listed below:

1. Inhibition of degradation pathways by MG-132 or Bafilomycin is expected to increase the concentration of most proteins in the cells, as proteins targeted for destruction will remain in the cell. For this reason, drug data needs to be corroborated with additional data before drawing any conclusions regarding BST-2 degradation pathways.

Even though in Figure 4C and 4D, we show that neither the total amount of the protein samples nor the amount of the RSV N protein is significantly affected by the treatments, we agree with the reviewer that data based on inhibitory drugs of degradation pathways alone are not sufficient to draw solid conclusions regarding BST-2 degradation pathways. To answer this question, we should use in the future specific siRNA to target lysosomal or proteasome pathway.

2. In Figure 3, the authors did not provide a detailed method for quantifying BST-2 in the plasma membrane and whole cell. The absence of a specific plasma membrane antibody is a major concern. This is particularly important in RSV-infected cells, where BST-2 staining is widely distributed.

We apologize for not being able to provide better labeling but we did not identify any functional antibodies specifically labeling the HEp-2 membrane. Moreover, WGA labeling showed a strong inner membrane background in these cells. A precise description of the methodology used is now included in the materials and methods section (lines 614-622).

Reviewer #3: PFU/ml are now provided in table S2. These results show that the effect of tetherin on virus production was analyzed under conditions resulting in a 2 log difference between WT RSV and Delta NS1 RSV titers. One could speculate that this pronounced difference in titers accounts for the differential tetherin sensitivity - thus it is conceivable that a mutant virus that replicates with markedly reduced efficiency as compared to the WT virus is also generally more sensitivity to inhibition by ISGs.

We agree with the remark, that's why we performed a distinct time point for the deltaNS1 virus at 72h. At this time point, the titer account is comparable to the WT and, moreover, the fold-change difference between knock down cells infected with RSV-mCherry or RSV-mCherry ΔNS1 was not only maintained but increased even more.

The data with vpu defective HIV-1 indicate that NS1 is not a tetherin antagonist. This is important information and should be shown in the manuscript. At the very least, the results should be stated and discussed in the manuscript.

The reviewer is right. NS1 alone is not a tetherin antagonist; NS1 participates in the mechanism developed by RSV to counteract BST2 restriction. Our results showed that, neither NS1 nor NS2 nor NS1 in combination with NS2 are able to antagonize BST2 restriction on HIV release, indicating that NS1 alone or in complex with NS2 are not sufficient to antagonist BST2 antiviral effect on HIV-1 release. This suggests that NS1 participates in BST2 countermeasure in the course of RSV infection that does not mimic those developed by HIV-1 Vpu to counteract BST2 restriction on HIV release. Our results also suggest that NS1 needs additional RSV proteins to counteract BST2 restriction.

This important information is now present in the new version, the figure corresponding to Vpu release has been added (Figure 8), and text modified (lines 359-365 in the results section and lines 507-509 in the discussion section). Moreover, we modified our comments when we first suggested that NS1 could be one of the BST2 antagonist (lines 36-37 in the abstract, lines 118-119 in the introduction and lines 504-509 in the discussion).

Part III – Minor Issues: Editorial and Data Presentation Modifications
Please use this section for editorial suggestions as well as relatively minor modifications of existing data that would enhance clarity.

Reviewer #1: None

Reviewer #2: Minor issues:

- The authors' one-step curve shown that the RSV cycle is around 24 hours in duration. Therefore, their multi-step curve has only two full cycles. More timepoints would be beneficial for Figure 1 clarity.

We thank the reviewer for the comment but in our practical experience and according to the literature (https://journals.asm.org/doi/10.1128/jvi.01693-10), RSV wt growth curve experiments in HEp-2 cells result in lower titers in the 72h timepoint compared to the 48h one (due to its cytopathic effect). Therefore, we do not believe an added time point in Figure 1 would provide further clarification in Figure 1. However, as request by the reviewer #3 (in the first review), we added the 72h timepoint in Figure 7C because the mutant virus replicates with reduced efficiency as compared to the WT virus.

- Figure S5 shows a 2.4-fold decrease in the total amount of BST-2 in Hep-2 cells infected by RSV. However, confocal images suggest that BST-2 migrates from the plasma membrane to the TGN. Upon visual inspection of the confocal images, it appears that the total amount of BST-2 remains constant, with only the location changing. The authors should verify if the time after infection is consistent between the confocal experiment and the Western Blot. Based on Figure 3, I would expect that at 24 hours post-infection, the total amount of BST-2 would be similar in non-infected and RSV-infected Hep-2 cells.

Western blot and immunofluorescence were both performed at 24h post infection (as indicated in the legend of the figures). We agree that upon visual inspection the total amount of BST2 seems to remain constant, but the intensity measured using Imaris Software as described in Materials and Methods, showed a reduced membrane intensity. Averages are shown below and all the raw data will be available in the manuscript.

	TGN (GM130) intensity	Membrane (Phalloidin) intensity
Non infected cells	28.301	19.267
RSV cells	32.757	11.097

- In my initial review, I did not mean to replace the bar graph with the FACS histogram (Figure 3A). I meant that the FACS histogram should be added to the figure along with the bar graph (or as supplemental figure).

We now added the FACS histogram as a supplementary data (S4 Fig)

Reviewer #3: (No Response)
